# The assessment of an inhibited, anxiety-prone temperament in a Dutch multi-ethnic population of preschool children

**DOI:** 10.1007/s00787-012-0299-0

**Published:** 2012-07-13

**Authors:** Leonie J. Vreeke, Peter Muris, Birgit Mayer, Jorg Huijding, Arjan E. R. Bos, Monique van der Veen, Hein Raat, Fop Verheij

**Affiliations:** 1 Institute of Psychology, Erasmus University Rotterdam, Burgemeester Oudlaan 50, Suite T12-35, Postbus 1738, 3000 DR Rotterdam, The Netherlands; 2 Clinical Psychology Science, Maastricht University, Maastricht, The Netherlands; 3 Faculty of Psychology, Open University, Heerlen, The Netherlands; 4 Ouder- en Kindzorg Rotterdam (Infant Welfare Center), Rotterdam, The Netherlands; 5 Department of Public Health, Erasmus Medical Center, Rotterdam, The Netherlands; 6 Department of Child and Adolescent Psychiatry, Erasmus Medical Center, Rotterdam, The Netherlands

**Keywords:** Behavioral inhibition, Parent-rating scale, Children, Psychometric properties, Anxiety

## Abstract

The Behavioral Inhibition Questionnaire-Short Form (BIQ-SF) is a 14-item parent-rating scale for assessing an inhibited, anxiety-prone temperament in preschool children. This study examined the psychometric properties of the BIQ-SF scores in a multi-ethnic community population of Dutch boys and girls aged 2.5–6 years (total *N* = 2,343, from which various subsamples were derived). Results revealed that the factor structure of the BIQ-SF was as hypothesized: a model with six correlated factors representing children’s inhibited behaviors in various social and non-social contexts provided a good fit for the data. The internal consistency of the BIQ-SF was generally satisfactory and scores on the scale were found to be fairly stable over a time period of up to 2 years. Parent-teacher agreement was acceptable, and relations between the BIQ-SF and observations of an inhibited temperament were moderate. Finally, BIQ-SF scores were positively associated with measures of anxiety and internalizing symptoms, whereas no significant links were found with externalizing symptoms. Altogether, these results provide support for the reliability and validity of the BIQ-SF as an economical method for assessing behavioral inhibition and anxiety proneness in young children.

## Introduction

Anxiety disorders are among the most prevalent psychiatric disorders in children and adolescents (e.g., [8]). However, it usually takes a long time before these children are referred to clinical services. As a result, anxiety problems tend to persist thereby having a fairly large impact on the lives of children before they receive treatment [47]. In order to minimize the adverse effects of anxiety problems on children’s lives, it is important to identify anxiety-prone and anxious children at a young age, so that prevention and intervention programs can be implemented as early as possible (e.g., [10, 40]).

One construct that seems particularly valuable for the early detection of anxiety-prone and anxious children is behavioral inhibition. This temperamental trait refers to the tendency to react with extreme shyness and withdrawal to novel objects, unknown situations, and unfamiliar people [29]. There is a growing body of evidence showing that behavioral inhibition indeed may be a prime risk factor for the development of anxiety problems (see for reviews: [15, 24]. An exemplary study is the longitudinal investigation by Biederman et al. [3], which demonstrated that inhibited preschool children were more likely to develop serious anxiety problems, including multiple anxiety disorders, separation anxiety disorder, social phobia, and agoraphobia over a 3-year period than a control group of non-inhibited children. As a result of these and other findings (e.g., [31, 39]), current models on the etiology of childhood anxiety disorders consistently include behavioral inhibition as an important vulnerability factor (e.g., [37]. Studies have indicated that behavioral inhibition is a genetically-based factor (e.g., [42]), that is normally distributed in the child population with about 15 % of the young people showing this temperament characteristic in the extreme [17, 27–29]. Most importantly, research has indicated that behavioral inhibition can be detected at a fairly young age, with some studies even documenting markers of this temperament factor in children as young as 4 months [30]. This underlines that behavioral inhibition is a highly relevant construct that can be useful for detecting vulnerable, anxiety-prone children at an early point during their development.

The assessment of behavioral inhibition has been typically confined to extensive laboratory procedures, during which features of the inhibited temperament (i.e., latency to approach, reluctancy to speech, and proximity to the parents) are observed while children are confronted with various unfamiliar social (e.g., an unknown peer or adult) and non-social (e.g., a black box or a novel computer game) stimuli (e.g., [26]. Although these lab observations certainly provide valuable information on children’s level of behavioral inhibition, they represent a rather time-consuming way of measuring this construct, and as such these procedures have limited utility for screening inhibited children in large community samples and longitudinal studies. For this reason, questionnaires such as the Short Temperament Scale for Children [44] and the Child Temperament Scale [46] have been employed to measure temperamental anxiety proneness in children. Although these scales have proven to be useful in this regard, it is also true that they measure a broader concept of temperament than behavioral inhibition. Meanwhile, a number of scales have been construed with a specific focus on behavioral inhibition (e.g., [18, 48], but these scales predominantly measure the social aspects of this temperament construct rather than inhibited behavior in response to a broad range of novel stimuli and situations [28].

A promising alternative might be the Behavioral Inhibition Questionnaire (BIQ; [4]), a 30-item parent-rating scale for assessing behavioral inhibition in six contexts: unfamiliar peers, unfamiliar adults, separation/preschool, physical challenging situations, performance situations, and unfamiliar situations in general. Studies examining the psychometric qualities of English and Dutch versions of the BIQ have generally yielded promising results [4, 5, 11, 34]. That is, support was found for the internal consistency of the scale, with most alphas for the total and subscales scores ranging between 0.70 and 0.90. Test–retest correlations indicated moderate stability for a time period of 12 months (*r*s between 0.58 and 0.79). Further, support was found for the hypothesized six-correlated factors structure of the scale, reflecting inhibited behavior in various specific contexts. In addition, agreement between mother and father reports of the BIQ was relatively good (*r*s between 0.69 and 0.84), while agreement between parent and teacher report appeared to be moderate (*r*s between 0.41 and 0.62). In addition, support was found for the validity of the scale: BIQ scores were positively correlated with related constructs as assessed with various child temperament questionnaires (*r*s between 0.78 and 0.89). Finally, it was found that the BIQ scores were low to moderately correlated with observational ratings of behavioral inhibition in a laboratory setting (*r*s between 0.25 and 0.46).

Interestingly, a shorter 14-item version of the scale, the BIQ-Short Form (BIQ-SF), has been construed, which of course has considerable potential for clinical, prevention as well as research purposes. So far, only one study has been conducted in which the reliability and validity of the BIQ-SF were examined [11]. Results indicated that the BIQ-SF has comparable psychometric properties as the full-length version. That is, support was found for the six-correlated factors structure of the scale, and the total and subscale scores show adequate internal consistency (with alphas ranging between 0.61 and 0.94), moderate 12-month test–retest reliability (*r*s between 0.57 and 0.76), and good validity as indicated by a strong correlation with the inverse score on the approach subscale of a general child temperament questionnaire (*r* = 0.87).

More research on the reliability and validity of the BIQ and BIQ-SF in multi-ethnic populations is needed, since previous studies have predominantly focused on children of Caucasian origin (i.e., >85 % in all studies; [4, 5, 11, 34]. Yet, research has demonstrated that children from various cultures display different levels of behavioral inhibition [7, 43], and there is also evidence from another Dutch investigation showing that children with an ethnic minority background run greater risk for developing anxiety disorders [22]. Thus, it would certainly be valuable to further explore the psychometric properties of this instrument in a more ethnically diverse group. Further, as previous studies have indicated that girls seem to be more anxiety-prone than boys [9], it seems also relevant to examine gender differences on this instrument.

With this in mind, the current study was set up to further examine the psychometric properties of the BIQ-SF in a large community sample of young Dutch children with a multi-ethnic background. The following aspects of the BIQ-SF were subjected to a psychometric evaluation: (a) the hypothesized six-correlated factors structure of the scale was tested by means of a confirmatory factor analysis, (b) various types of reliability were investigated including the internal consistency, test–retest reliability, and cross-informant agreement, and (c) several aspects of the validity were explored such as the relations with anxiety and internalizing (i.e., convergent validity) and externalizing (i.e., divergent validity) symptoms as well as the relations between BIQ-SF scores of parents and teachers and laboratory observations of an inhibited temperament (i.e., predictive validity). Further, (d) gender and ethnic differences in behavioral inhibition as indexed by the BIQ-SF were explored.

## Method

### Participants and procedure

Parents (in most cases the mother) of 2,343 2.5 to 6-year-old non-clinical children (*M* = 3.59, SD = 0.77; 1,189 boys and 1,147 girls)^1^ completed the BIQ-SF. More than two-third of the parents (*N* = 1,636) were visiting the infant welfare center in Rotterdam, the Netherlands, and participated in a longitudinal study on the relation between behavioral inhibition and anxiety in young children. Other parents were recruited via playgroups and a mailing of the local council in Gouda and Woerden, two smaller cities in the vicinity of Rotterdam. The ethnic background of this sample was mixed: 65 % was from original Dutch descent, 6 % had a Surinam, 6 % a Moroccan, 4 % a Turkish, 3 % an Antillean, and 16 % another ethnic background. No exact information about socioeconomic status was available, but in the Netherlands a non-Dutch ethnicity is usually indicative for a lower SES. To assess the temporal stability and validity of the BIQ-SF, 1,636 parents were contacted again 1 year later, and asked to complete the BIQ-SF for a second time, along with a set of other questionnaires. Of the parents in our longitudinal study, 70 were moved or could not be contacted again, 94 responded to our mailing stating explicitly that they were not willing to participate again, while 740 parents did not respond at all to our mailing. Thus, almost half of the parents eventually agreed to participate in this follow-up assessment (*n* = 732; 45 %). To examine selection bias, BIQ-SF scores of parents who did and did not participate in this follow-up assessment were compared, but no significant differences were found. Due to missing values, data of 716 children were used to explore the test–retest stability and validity of the BIQ-SF (371 boys and 345 girls). These children received a small present for their parents’ participation (e.g., a sticker book). Another year later, the 732 parents were approached again with the request to fill out the BIQ-SF for a third time to assess the test–retest stability after 2 years. At this time, parents of 284 children (153 boys and 131 girls; 39 %) agreed to participate. Again, parents who participated in this 2-year follow up did not differ from the non-participating parents on any of the BIQ-SF scores. A randomly selected subsample of these children (*n* = 184; 97 boys and 87 girls; 65 %) attended an individual assessment of approximately thirty minutes at the university laboratory to assess their temperament by means of behavioral observations (see below). Finally, teachers of the 184 children participating in the behavioral observations were also contacted and asked to complete the BIQ-SF as well as a standardized rating scale for measuring anxiety symptoms. One-hundred and twenty-two teachers (i.e., 67 %) responded positively to this request and returned the materials to the researchers. Table 1 provides a schematic overview of the participants and procedures during each assessment occasion of this study.

**Table 1:**
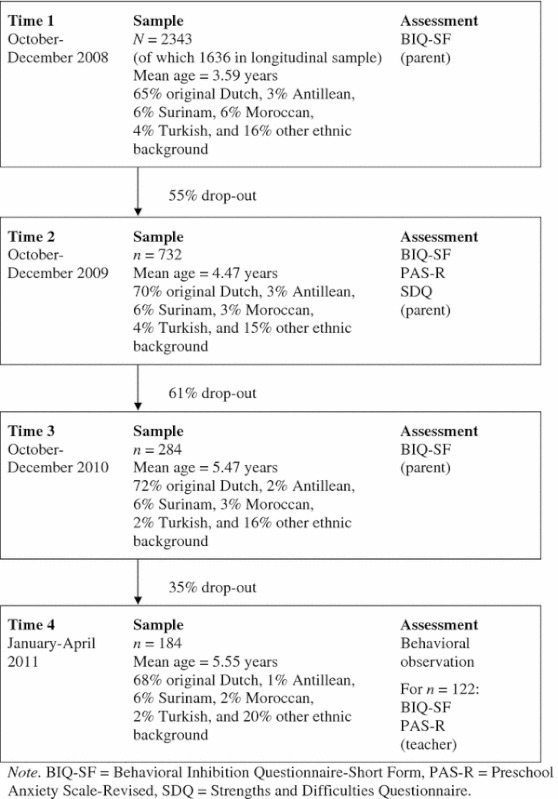
Schematic overview of the present study

### Questionnaires

The Behavioral Inhibition Questionnaire-Short Form (BIQ-SF; [11] is an abbreviated 14-item version of the original BIQ [4], which is a parent-report instrument assessing behavioral inhibition in six contexts: unfamiliar peers (3 items; e.g., ‘My child is shy when first meeting new children’), unfamiliar adults (2 items; e.g., ‘My child is very quiet with adult strangers’), performing in front of others (2 items; e.g., ‘My child dislikes being the centre of attention’), preschool/separation (2 items; e.g., ‘My child gets upset at being left in new situations for the first time, e.g., kindergarten’), unfamiliar situations (2 items; e.g., ‘My child approaches new situations or activities very hesitantly’), and physical challenges (2 items; e.g., ‘My child is hesitant to explore new play equipment’). Parents answer the questions on a 6-point Likert scale, ranging from 1 (*hardly ever*) to 6 (*almost always*). BIQ-SF total scores are calculated by summing the scores on all items (range 14–84), with higher scores being indicative for higher levels of inhibited behavior. In addition, subscale scores can be computed by summing across relevant items. As already noted in the introduction, the psychometric properties of the original BIQ are good [4, 5, 34], and there is also tentative support for the reliability and validity of the BIQ-SF [11].

The Preschool Anxiety Scale-Revised (PAS-R; [12]) is an adaptation of the Preschool Anxiety Scale (PAS; [45]), a 30-item parent-based questionnaire assessing anxiety disorder symptoms in young children. More specifically, the PAS-R measures symptoms of social anxiety disorder (7 items; e.g., ‘My child is afraid to go up to a group of children to join their activities’), separation anxiety disorder (5 items; e.g., ‘My child would be upset at sleeping away from home’), generalized anxiety disorder (7 items; e.g., ‘My child has difficulty stopping him/herself from worrying’), specific fears (i.e., specific phobia; 9 items; e.g., ‘My child is frightened of dogs’), and obsessive–compulsive disorder (2 items; e.g., ‘My child becomes distressed by thoughts or images in his/her head’). Items are rated by parents on a 5-point scale ranging from 0 (*not at all true*) to 4 (*very often true*). PAS-R total scores can be calculated by summing the scores on all items (range 0–120), and subscale scores are computed by summing relevant items. Adequate internal consistency, test–retest reliability, cross-informant reliability, and construct validity for the scale have been demonstrated [12, 45], with the reliability of the obsessive–compulsive scale being somewhat lower than for the other scales.

The Strengths and Difficulties Questionnaire (SDQ; [19, 20] is a 25-item parent-rating scale of emotional and behavioral problems as well as abilities and strengths in children aged 3–16 years. The SDQ consists of five scales of five items each, generating scores for conduct problems (e.g., ‘Steals from home, school or elsewhere’), hyperactivity-inattention (e.g., ‘Restless, overactive, cannot stay still for long’), emotional symptoms (e.g., ‘Many worries, often seems worried’), peer problems (e.g., ‘Rather solitary, tends to play alone’), and prosocial behavior (e.g., ‘Helpful if someone is hurt, upset or feeling ill’). Items are scored on a 3-point scale from 0 (*not true*) to 2 (*certainly true*) indicating how well they correspond to the child’s behavior during the past 6 months. Subscale scores can be computed by summing across items after recoding a number of reversed items. In the present study, the emotional symptoms and peer problems subscales of the SDQ were combined into an ‘internalizing’ subscale, whereas the conduct and hyperactivity-inattention subscales were joined into an ‘externalizing’ subscale (range from 0 to 20; [21]). Satisfactory psychometric properties of the SDQ have been reported in various studies (e.g., [19, 20, 38, 50]).

The teacher versions of the BIQ-SF and the PAS-R were identical to the scales as completed by the parents, except that instructions and items were rephrased in terms of the teacher’s perspective (e.g., “This child dislikes being the centre of attention”). Good psychometric properties have also been documented for the teacher version of the 30-item BIQ [4, 11, 34].

It should be mentioned that the PAS-R and the SDQ proved to be reliable in terms of internal consistency. That is, Cronbach’s alphas for various (sub)scales were well above the 0.70, except for the obsessive compulsive subscale of the PAS-R (alphas being 0.38 and 0.58 for the parent and teacher version, respectively), a result which is in line with previous studies.

### Behavioral observations

A number of behavioral tasks, based on the procedures used by Asendorpf [1], Kagan et al. [28], Bishop et al. [4], Edwards [11], and Van Brakel et al. [49], were used in order to assess observable manifestations of the inhibited temperament. Parents and children were observed during a lab assessment that included both social and non-social tasks. A standardized protocol (see Table 2) was used that was carried out by four trained master students in psychology (all female). Children were videotaped so that it was possible to score their behavior afterwards, and to be able to compute the interrater reliability for the various behavioral inhibition indexes. For this purpose, two other master psychology students were extensively trained by the principal investigator in coding these behaviors. Training included a review of the coding manual, observation of videotaped examples of the behaviors being coded, and coding practice until consistency was reached. Coders were blind to the BIQ-SF scores of the children. Interrater reliability was calculated with single-measure intraclass correlations (ICCs) coefficients. The following variables were coded across these tasks: number of encouragements (ICC = 0.85), latency of speech (ICC = 0.80), and latency to approach (ICC = 0.93). In addition, the overall level of behavioral inhibition across these tasks was scored on a 10-point scale, ranging from 1 (*extremely uninhibited*) to 10 (*extremely inhibited*) (ICC = 0.80). For statistical analysis, all the measures were averaged across the different observers for each child. A more detailed description of the lab assessment and the coding procedures are available from the first author.

**Table 2:** Brief description of various episodes during the observation procedure

Episode	
Opening questions	The child is asked a series of simple, standardized questions (e.g., ‘What’s the name of your school?’, ‘Do you have many friends at school?’) by the first experimenter in the presence of the mother.
Separation	After these questions, the mother is asked to leave the observation room.
Three minutes alone	The first experimenter also leaves the room quickly after mother’s departure, and the child is left alone with some toys in the room for three minutes.
Stranger	Thereafter, an unfamiliar female student enters the room, and starts to build a tower of wooden blocks without making any contact with the child. If the child does not approach spontaneously, after 1 min, the student starts to invite him or her to join in.
Singing a song	The unfamiliar student leaves the room, and the first experimenter returns with a big winnie the pooh wearing a birthday hat. The experimenter asks the child to sing a birthday song because it is Pooh bear’s birthday.
Throw a ball	The child is asked to throw a ball in a basket from a distance at his/her preference.
Jumping	The child is asked to fall as straight as possible forward and backward on a big mattress.
Black boxes	The child is asked to feel in two closed black boxes and has to guess what is inside by feeling with his/her hand.
Mystery Guest	An unfamiliar person (i.e., a third female student) wearing a cape, a mask, and a wig enters the room. The child is asked to “unmask” this so-called mystery guest in order to check the identity of this person.

The ethical committee of psychology of Erasmus University provided official approval for this observation procedure.

### Data analysis

Confirmatory factor analysis was performed using analysis of moment structures (AMOS; version 17) to estimate how well the proposed model (i.e., a six-correlated factors model) as found in the previous study by Edwards [11] fitted the Dutch BIQ-SF. The estimation method employed was maximum likelihood. As the likelihood ratio Chi-square (χ^2^) is often large and significant in large samples, other indices are generally used to test the goodness-of-fit of a model: the Comparative Fit Index (CFI), the goodness-of-fit index (GFI), Tucker Lewis Index (TLI), and the root mean square error of approximation (RMSEA; [6]. The possible values of the CFI, GFI, and TLI range between 0 and 1, with values >0.95 indicating good fit. For the RMSEA, smaller values indicate a better fit, with values <0.08 being indicative of a satisfactory model fit [25].

Further, Cronbach’s alphas were computed to determine the internal consistency of the total and subscale scores for both the parent and teacher versions of the BIQ-SF. Gender differences in total and subscale scores of the parent and teacher BIQ-SF were investigated by means of a multivariate analysis of variance (i.e., MANOVA). A MANOVA was also performed to test differences in BIQ-SF scores among Dutch children and children from other ethnic decent. To assess the test–retest reliability of the BIQ-SF, 1 and 2-year Pearson correlation coefficients were calculated for the parent BIQ-SF. Further, single-measure intraclass correlations (ICC) coefficients were calculated between parent and teacher reports of the BIQ-SF to examine the cross-informant reliability.

For the parent report, convergent and divergent validity were evaluated by calculating Pearson correlation coefficients between behavioral inhibition on the one hand, and symptoms of anxiety, internalizing and externalizing symptoms on the other hand. For the teacher version of the BIQ-SF, convergent validity was assessed by calculating Pearson correlation coefficients between behavioral inhibition and teacher reported symptoms of anxiety.

Finally, as a test of the predictive validity of the BIQ-SF, parent and teacher reports of behavioral inhibition were related to direct observations of specific behaviors that have been shown to be indicative of an inhibited temperament (e.g., [1, 23, 49].

## Results

### Confirmatory factor analysis

A confirmatory factor analysis was performed to test the fit of the hypothesized six-correlated factors model.^2^ The goodness-of-fit indices for this model were as follows: RMSEA = 0.05, CFI = 0.98, GFI = 0.97, and TLI = 0.96, which indicates that this model provided a good fit for the data in this Dutch multi-ethnic sample. Highly comparable results were obtained when analyzing the data of native Dutch children (RMSEA = 0.06, CFI = 0.98, GFI = 0.97, and TLI = 0.96) and children with another ethnic background (RMSEA = 0.06, CFI = 0.97, GFI = 0.97, and TLI = 0.95) separately. For the teacher version of the BIQ-SF, the six-correlated factors model also yielded a satisfactory fit, with RMSEA = 0.01, CFI = 0.99, GFI = 0.98, and TLI = 0.99. Standardized item loadings for this model as obtained for the parent and teacher version of the BIQ-SF are presented in Table 3. Correlations among factors were positive and ranged between 0.37 and 0.83 for the parent version and between 0.59 and 0.85 for the teacher version. We also explored whether the covariance among the six factors can be explained by a single higher-order factor of behavioral inhibition (see [4, 34]. This ‘six-correlated factors loading onto 1 higher-order factor model’ also provided a satisfactory fit for the parent (RMSEA = 0.06, CFI = 0.97, GFI = 0.97, TLI = 0.97) and teacher version (RMSEA = 0.02, CFI = 0.92, GFI = 0.95, TLI = 0.95) of the BIQ-SF.

**Table 3:** Results of the confirmatory factor analysis: Standardized factor loadings as obtained for the six-correlated factors model for the parent (*N* = 2,343) and teacher version (*n* = 122) of the BIQ

Factor	Items (abbreviated)	I	II	III	IV	V	VI
Peers	Shy when first meeting new children	0.81 (0.79)					
	Approaching a group of children and join in	0.86 (0.92)					
	Watching other children rather than join	0.81 (0.84)					
Physical challenge	Cautious in activities involving physical challenge		0.68 (0.78)				
	Hesistant to explore new play equipment		0.71 (0.81)				
Preschool/separation	Upset when left alone in new situation			0.80 (0.74)			
	Takes many days to adjust to new situations			0.88 (0.94)			
Performance situations	Dislikes being centre of attention				0.84 (0.74)		
	Reluctant to perform in front of others				0.75 (0.94)		
Unfamiliar adults	Quiet around new (adult) guests					0.90 (0.95)	
	Quiet with adult strangers					0.94 (0.92)	
Unfamiliar situations	Hesitant in approaching new situations or activities						0.80 (0.90)
	Clingy in homes of unknown people						0.76 (0.78)
	Nervous or uncomfortable in new situations						0.79 (0.89)

Data for the teacher version are displayed in parentheses

*BIQ-SF* Behavioral Inhibition Questionnaire-Short Form

### Reliability

Mean scores (standard deviations) and reliability indices of the parent and teacher versions of the BIQ-SF are presented in Table 4. As can be seen, the Cronbach’s alphas of the parent-version of the BIQ-SF were 0.92 for total score and between 0.77 and 0.91 for various subscales, with only the physical challenges subscale BIQ-SF being somewhat lower (α = 0.61). These results indicate good internal consistency for the total score and various subscales, with the exception of the physical subscale. For the teacher report, Cronbach’s alphas of the BIQ-SF total score (α = 0.95) and various subscales (α’s between 0.72 and 0.94) were highly comparable.

**Table 4:** Mean scores (standard deviations) and reliability indices for the parent and teacher versions of the BIQ-SF

	Parent				Teacher		
	*M* (SD) (*N* = 2,343)	*α* (*N* = 2,343)	*r*1 (*n* = 716)	*r*2 (*n* = 284)	*M* (SD) (*n* = 122)	*α* (*n* = 122)	Parent/teacher ICC (*n* = 122)
BIQ-SF							
Total score	34.42 (11.72)	0.92	0.73**	0.65**	31.47 (12.84)	0.95	0.40**
Peers	7.98 (3.32)	0.86	0.66**	0.60**	6.86 (3.09)	0.89	0.34**
Physical challenges	3.82 (1.91)	0.61	0.56**	0.36**	3.82 (1.98)	0.72	0.25**
Preschool/separation	4.59 (2.22)	0.82	0.56**	0.54**	4.06 (1.90)	0.80	0.22*
Performance situations	4.94 (2.26)	0.77	0.59**	0.52**	5.13 (2.46)	0.88	0.35**
Unfamiliar adults	5.05 (2.38)	0.91	0.60**	0.58**	4.90 (2.49)	0.94	0.41**
Unfamiliar situations	8.01 (2.98)	0.81	0.65**	0.54**	6.72 (3.27)	0.89	0.23*

*BIQ-SF* Behavioral Inhibition Questionnaire-Short Form. *r*1 1-year test–retest correlation, *r*2 2-years test–retest correlation, *ICC* intraclass correlations

^*^ *p* < .05, ^**^ *p* < .01

Further, the 12-month correlations of the BIQ-SF scales (parent report) indicated moderate stability (*r*s between 0.56 and 0.73, *p*s < 0.01). Correlations were somewhat lower over the 24-month period (*r*s between 0.36 and 0.65, *p*s < .01).

The correspondence between parent and teacher BIQ–SF total scores was moderate (ICC = 0.40, *p* < .01). The cross-informant agreement for various subscales was in a similar range (ICC’s between 0.22 and 0.41, *p*s < .05).

### Convergent and divergent validity

Table 5 shows the correlations between parent- and teacher-reported BIQ-SF scales on the one hand, and the PAS-R and SDQ scores on the other hand. As can be seen, significant and positive correlations were found between the BIQ-SF total and subscale scores and anxiety scores as obtained with the PAS-R. Note also that this was true for the parent as well as the teacher version of the BIQ-SF. Tests for comparing correlated correlation coefficients [36] showed that the links with anxiety symptoms were stronger for the teacher report than for the parent version of the BIQ-SF (with exception of the peers subscale all *t*s (838) ≥2.91, *p*s < .01). Further, for the parent version, BIQ-SF total and subscale scores were found to be significantly and positively associated with the internalizing subscale of the SDQ (*r*s between 0.27 and 0.42, *p*s < .01), whereas non-significant relations were observed with the externalizing subscale of the SDQ. The only exceptions were the BIQ-SF preschool/separation and the unfamiliar situations subscales, which displayed small, but significant positive associations with the SDQ externalizing subscale.

**Table 5:** Correlations between the various BIQ-SF scales on the one hand, and PAS-R and SDQ scales on the other hand

	Parent (*n* = 716)			Teacher (*n* = 122)
	PAS-R	SDQ internalizing	SDQ externalizing	PAS-R
BIQ-SF				
Total score	0.67**	0.42**	0.05	0.80**
Peers	0.53**	0.37**	−0.01	0.61**
Physical challenges	0.38**	0.26**	−0.03	0.61**
Preschool/separation	0.57**	0.36**	0.13**	0.69**
Performance situations	0.46**	0.29**	−0.01	0.63**
Unfamiliar adults	0.49**	0.27**	0.06	0.72**
Unfamiliar situations	0.63**	0.38**	0.09*	0.81**

*BIQ-SF* Behavioral Inhibition Questionnaire-Short Form, *PAS-R* Preschool Anxiety Scale-Revised, *SDQ* Strenghts and Difficulties Questionnaire

^*^ *p* < .05, ^**^ *p* < .01

### Predictive validity: relations with observation measures of behavioral inhibition

Correlations between the BIQ-SF total score and behavioral observations of the children’s inhibited temperament are shown in Table 6. All correlations between the behavioral observations and questionnaire scores were in the low to moderate range, but nonetheless in the expected direction. That is, parent report of behavioral inhibition was positively and significantly related to latency of speech, number of encouragements, and latency to approach, as well as to the overall observational measure of behavioral inhibition (*r*s between 0.18 and 0.24). The teacher version of the BIQ-SF was only significantly related to the overall observational measure of behavioral inhibition and latency to approach (*r*s being 0.25 and 0.27, respectively), while no significant correlations were observed with latency of speech and number of encouragements (*r*s being 0.06 and 0.15, respectively).

**Table 6:** Correlations between the BIQ-SF of parents and teachers and indices of an inhibited temperament

	Parent BIQ-SF (*n* = 184)	Teacher BIQ-SF (*n* = 122)
Observer ratings of BI	0.24**	0.25**
Latency of speech	0.19**	0.06
Number of encouragements	0.18*	0.15
Latency to approach	0.20**	0.27**

*BI* behavioral inhibition, *BIQ-SF* Behavioral Inhibition Questionnaire-Short Form

^*^ *p* < .05, ^**^ *p* < .01

### Gender and ethnic differences

Gender differences were examined for the parent and teacher versions of the BIQ-SF by means of a MANOVA. For the parent report, boys and girls displayed comparable BIQ-SF total scores, but on the subscales some gender differences were observed. That is, parents rated boys as significantly more inhibited on the performance subscale [means being 5.14 (SD = 2.29) versus 4.72 (SD = 2.18); *F*(2,2336) = 19.77, *p* < .001, η^2^ = 0.008], whereas girls were scored as significantly more inhibited when meeting unfamiliar adults [means being 4.91 (SD = 2.37) vs. 5.20 (SD = 2.38); *F*(2,2336) = 8.34, *p* < .01, η^2^ = 0.004]. For teacher report, no significant gender differences were observed.

A MANOVA was also carried out to evaluate differences on BIQ-SF scales among the most sizable ethnic groups in this study (i.e., original Dutch, Surinam, Moroccan, Turkish, and Antillean). No significant differences between these groups were observed for the BIQ-SF total score and most of the subscales. However, on the unfamiliar situations subscale, a significant difference was found [*F*(4,2132) = 3.24, *p* < .05, η^2^ = 0.007]. Post hoc tests (which controlled for unequal sample sizes) indicated that Turkish children scored significantly lower on this subscale as compared to the other groups [means being 6.91 (SD = 2.67) for Turkish vs. 8.05 (SD = 2.94) for Dutch, 8.23 (SD = 2.97) for Surinam, 8.03 (SD = 3.40) for Moroccan, and 8.11 (SD = 3.03) for Antillean children (all *p*s < .05)].

## Discussion

The aim of this study was to examine the reliability and validity of a parent and teacher rating questionnaire for measuring an inhibited, anxiety-prone temperament in young children. Results were largely in line with the findings of previous research [4, 5, 11, 34], and indicate that the BIQ-SF has good psychometric qualities. Confirmatory factor analysis provided support for a model of six-correlated factors, which reflects the intended subscales of peers, physical challenging situations, preschool/separation, performance situations, unfamiliar adults, and unfamiliar situations [11]. Moreover, a higher-order model also provided a satisfactory explanation for the covariance among the six first-order factors, which justifies the employment of the BIQ-SF total scale.

The internal consistency of the total BIQ-SF and subscale scores was found to be moderate to good, and this was true for both the teacher and the parent version. Further, test–retest correlations of the BIQ-SF over 12 and 24 months indicated that behavioral inhibition scores were fairly stable across time. The magnitude of the correlations was comparable with those reported in previous studies on the test–retest stability of the BIQ and the BIQ-SF, with longitudinal data on behavioral inhibition showing that this temperamental trait may change during early childhood, but still is an enduring characteristic in most of the children [16, 41].

This study also provides support for the convergent and divergent validity of the BIQ-SF. That is, substantial and positive correlations were found between BIQ total and subscale scores and scores on the PAS-R (parent and teacher report), a questionnaire assessing anxiety disorder symptoms in young children, and the SDQ internalizing subscale (parent report), whereas small and mostly non-significant correlations were found between the BIQ-SF and the SDQ externalizing subscale (see for a similar result: [5]. Further, significant correlations were found between the BIQ-SF (parents and teachers report), and observable indicators this temperamental trait such as latency of speech, number of encouragements needed, and latency to approach during various behavioral tasks. It was found that the parent version of the BIQ-SF was more strongly correlated with observational indices of behavioral inhibition than the teacher version, probably because parents are able to observe their child responding to a broader range of stimuli and situations in daily life, and therefore may have a slightly better impression of their child’s inhibited behavior. Admittedly, correlations between BIQ-SF scores and observational measures were rather modest, which is in line with previous research investigating the link between questionnaire and observational data of behavioral inhibition in children (e.g., [4, 17, 34, 49]. This is most likely due to the fact that questionnaire items are formulated in a more general way thereby covering a wide range of inhibited behaviors, whereas the behavioral observations are carried out in a number of specific, non-naturalistic situations. Further, as noted by Epstein [13, 14], the sampling error is large when observing temperamental traits on only one occasion. Such traits can be more reliably assessed when the pertinent behavior is observed and averaged across a wide range of events. Behavior observed on a single occasion provides a rather limited sample of the child’s behavior, which is strongly linked to that particular situation and difficult to generalize to other contexts. Finally, previous studies examining the relations between behavioral inhibition and observational measures also demonstrated that there are marked individual differences in maternal accuracy when predicting child behavior [32, 33].

In the present study, few gender differences in behavioral inhibition were documented, which is in keeping with previous studies (e.g., [17] and indicates that this temperamental trait is equally relevant for boys and girls. Nevertheless, on two BIQ-SF subscales a significant gender difference did emerge. First, consistent with findings by Edwards [11], Broeren and Muris [5], and Kim et al. [34], parents rated boys as significantly more inhibited in performance situations than girls. In contrast, girls were rated as significantly more inhibited when meeting unfamiliar adults, a result which is in line with a study by Kochanska [35]. Although the findings suggest that there are gender differences in behavioral inhibition between boys and girls depending on the situational context, it should also be noted that these gender differences were small in absolute terms and mainly emerged as a result of the large sample size, and thus may have relatively little practical value.

This study made use of a large multi-ethnic population and so we were able to explore differences in behavioral inhibition across original (Caucasian) Dutch children and children with a Surinam, a Moroccan, a Turkish, and an Antillean background. In general, results indicated that all ethnic subgroups displayed comparable levels of behavioral inhibition. The only difference was that Turkish children scored significantly lower on the unfamiliar situations subscale of the BIQ-SF as compared to the other ethnic groups. Interestingly, this finding is in contrast with a finding by Bengi-Arslan et al. [2], who observed that parents of Turkish children scored their offspring as higher on anxiety as compared to other Dutch peers. Thus, while Turkish children are scored lower on an inhibited, anxiety-prone temperament, they appear to display higher levels of anxiety problems. This apparently contradictory result can be explained in various ways. On the one hand, it could be speculated that parents of Turkish children provide lower scores on the unfamiliar situations subscales because of cultural differences in the perception and expectations of children’s behaviors when confronting such events. Otherwise, it should also be kept in mind that anxiety pathology is determined by multiple factors [37, 51], which means that behavioral inhibition is only one of the multiple vulnerability and risk factors. Thus, an anxiety problem may even arise without the presence of an inhibited temperament.

Several limitations of this study should be mentioned. First, the convergent validity of the BIQ-SF was examined through its relations with questionnaires for measuring anxiety and internalizing symptoms. However, it would be useful to validate the scale against an alternative instrument for measuring children’s inhibited temperament. Second, the attrition rates in this study were substantial, which of course introduces the possibility of a selection bias. However, statistical analyses could not detect differences in levels of behavioral inhibition scores between children of whom parents continued to participate in the study versus children of parents who dropped out. Third and finally, the teacher sample was rather small, although the psychometric properties of the teacher version of the BIQ-SF were highly comparable to those obtained for the parent version. Besides these limitations, several strengths of this study should also be mentioned. First, part of this study relied on a longitudinal design with a large, multi-ethnic sample. In addition, this study relied on both multi-informant questionnaire data as well as a behavioral observation.

To conclude, the results of this study indicate that the BIQ-SF has good psychometric properties. With only 14 items, this instrument provides a reliable, valid and economical method for assessing inhibition and anxiety proneness in children at a fairly young age. Early detection of vulnerable youth makes it possible to implement prevention programs, which focus on the elimination of avoidance behavior in children and anxious and overprotective rearing styles of parents.

## Footnotes

1 Parents of seven children did not report the gender of their child.

2 Following the suggestion of an anonymous reviewer, an exploratory factor analysis (i.e., principal components analysis with oblimin rotation) was also performed on the BIQ-SF data. This analysis yielded three components with an eigenvalue >1, which accounted for 63.71 % of the total variance. The first component consisted of items belonging to the performance situations, unfamiliar adults, unfamiliar peers, and unfamiliar situations subscales, the second component only contained items of the physical challenges subscale, while the third component was composed of items of the preschool/separation subscale.
